# Retraction: Impact of Tight Collars and Neckties on Cervical Dysfunction Among Corporate Workers

**DOI:** 10.7759/cureus.r254

**Published:** 2026-10-21

**Authors:** Anjum S Inamdar, Suraj Kanase

**Affiliations:** 1 Department of Physiotherapy, Krishna College of Physiotherapy, Krishna Vishwa Vidyapeeth (Deemed to be University), Karad, IND; 2 Department of Neurosciences Physiotherapy, Krishna College of Physiotherapy, Krishna Vishwa Vidyapeeth (Deemed to be University), Karad, IND

This article has been retracted by the Editor-in-Chief. After publication, concerns were raised about the data reported in this article. A review of the original study data found that the data collection period and the study population differ from those described in the article, and that the reported statistical results could not be reproduced. The authors disagree with the decision to retract.

